# Surgery for fistula-in-ano in a specialist colorectal unit: a critical appraisal

**DOI:** 10.1186/1471-230X-11-120

**Published:** 2011-11-09

**Authors:** Pierpaolo Sileri, Federica Cadeddu, Stefano D'Ugo, Luana Franceschilli, Giovanna Del Vecchio Blanco, Elisabetta De Luca, Emma Calabrese, Sara Mara Capperucci, Valeria Fiaschetti, Giovanni Milito, Achille Lucio Gaspari

**Affiliations:** 1 Department of Surgery, University Hospital Tor Vergata, Rome, Italy; 2 Department of Radiology, University Hospital Tor Vergata, Rome, Italy; 3 Department of Gastroenterology, University Hospital Tor Vergata, Rome, Italy

## Abstract

**Background:**

Several techniques have been described for the management of fistula-in-ano, but all carry their own risks of recurrence and incontinence. We conducted a prospective study to assess type of presentation, treatment strategy and outcome over a 5-year period.

**Methods:**

Between 1st January 2005 and 31st March 2011 247 patients presenting with anal fistulas were treated at the University Hospital Tor Vergata and were included in the present prospective study. Mean age was 47 years (range 16-76 years); minimum follow-up period was 6 months (mean 40, range 6-74 months).

Patients were treated using 4 operative approaches: fistulotomy, fistulectomy, seton placement and rectal advancement flap. Data analyzed included: age, gender, type of fistula, operative intervention, healing rate, postoperative complications, reinterventions and recurrence.

**Results:**

Etiologies of fistulas were cryptoglandular (n = 218), Crohn's disease (n = 26) and Ulcerative Colitis (n = 3). Fistulae were classified as simple -intersphincteric 57 (23%), low transphincteric 28 (11%) and complex -high transphicteric 122 (49%), suprasphincteric 2 (0.8%), extrasphinteric 2 (0.8%), recto-vaginal 7 (2.8%) Crohn 26 (10%) and UC 3 (1.2%).

The most common surgical procedure was the placement of seton (62%), usually applied in case of complex fistulae and Crohn's patients.

Eighty-five patients (34%) underwent fistulotomy, mainly for intersphincteric and mid/low transphincteric tracts. Crohn's patients were submitted to placement of one or more loose setons.

The main treatment successfully eradicated the primary fistula tract in 151/247 patients (61%). Three cases of major incontinence (1.3%) were detected during the follow-up period; Furthermore, three patients complained minor incontinence that was successfully treated by biofeedback and permacol injection into the internal anal sphincter.

**Conclusions:**

This prospective audit demonstrates an high proportion of complex anal fistulae treated by seton placement that was the most common surgical technique adopted to treat our patients as a first line. Nevertheless, a good outcome was achieved in the majority of patients with a limited rate of faecal incontinence (6/247 = 2.4%). New technologies provide promising alternatives to traditional methods of management particularly in case of complex fistulas. There is, however, a real need for high-quality randomized control trials to evaluate the different surgical and non surgical treatment options.

## Background

Anal fistula represents an important aspect of colorectal practice, being a distressing condition for the patient and sometimes a challenge for the surgeon.

The majority of anal fistulae are of crypto-glandular origin, following anorectal abscess in 7-40% of cases [1]. Besides, anal fistulae are sometimes associated to other conditions, mainly inflammatory bowel disease, particularly Crohn's disease.

According to the cryptoglandular hypothesis, intersphinteric gland infection is the initiating event in the formation of perianal fistulas [2]. The sepsis arising within these glands can spread into the inter-sphincteric space, and from here towards the different anorectal planes causing abscesses and fistulae. Parks suggested the most widely used classification of intersphincteric, transphincteric, suprasphincteric, and extrasphincteric fistulas [3].

The targets of surgical management are sepsis drainage and fistula tracts removal, preserving sphincter integrity whenever possible and avoiding recurrence of sepsis.

A high success rate is generally reported in literature for low transphincteric fistulas involving the lower 3^rd ^of the external anal sphincter [4].

Besides, the treatment of complex anal fistulas is still a challenge for the colorectal surgeon with variable success rate reported in different trials [4,5].

Surgical procedures for high transphincteric fistulas include advancement flap closure, with a different success rate according to the etiology of the fistula [6-8] and a recurrence rate ranging between 0% and 63%. Cutting setons have been used in an attempt to slowly divide the sphincters while allowing scarring to occur and limit disruption of the muscular ring, with recurrence rates from 22% to 39% [9,10]. During the last ten years, fibrin glue injection has become a popular alternative to the cutting seton and mucosal advancement flap repair of complex fistulas; however, the published success rates widely between 14% and 60% [11-13].

The aim of the present study was to assess prospectively the presentation, classification, management and outcome of a series of 247 consecutive patients presenting with fistula-in-ano at our institution in a 5-year period.

## Methods

Between January 2005 and March 2011, 247 patients underwent Examination Under Anaesthesia (EUA) for fistula-in-ano at our Institution and were included in this prospective study.

All patients were examined by a colorectal surgeon in the outpatients clinic; the pre-treatment evaluation included anamnesis, concerning pregnancies, episiotomy, previous gynaecological, urological, or ano-rectal surgery and symptoms, clinical examination of the perineum and anorectum and proctoscopy.

The Wexner continence score and the FISI score were adopted to evaluate the degree of continence in every patient.

Colonoscopy, anorectal manometry, magnetic resonance and/or endoanal ultrasonography were performed if necessary, particularly in patients with diagnosis of inflammatory bowel disease.

Fistulae were classified on the basis of operative findings according to Parks' classification. Written informed consent had been obtained from all the subjects after a full explanation of the procedure. All surgical procedures were performed by four certified colorectal surgeons (PS, GM, FC, LF).

Surgery was performed with the patient in lithotomy position under local anaesthesia in most cases and, if necessary, general anaesthesia was provided. The standard preoperative protocol included a phosphate enema performed 12 hours before surgery and 500 mg of metronidazole plus 2 gr of cefotaxime given intravenously at the beginning of surgery.

After the discharge, patients were assessed at the first follow up visit after 7 days; further controls were scheduled at 1, 3 and 6 months. Additional controls were performed to manage the cutting setons. Thereafter phone interviews were performed annually.

All the data concerning baseline characteristics of patients, details of presentation, fistula etiology and anatomy, surgery performed and surgical outcomes were analyzed.

During the follow up period details of wound healing, postoperative complications (bleeding, nausea, vomiting, urinary dysfunction) and time of resumption of work, were recorded; late complications such as fistula recurrences, flatus or liquid incontinence, reinterventions were also assessed.

## Results

Between January 2005 and March 2011, 247 patients presented with anal fistula and were treated at the Department of Surgery, Tor Vergata University Hospital, Rome.

One hundred forty-nine were males and 98 females; mean age was 47 years (range 16-76 years). Mean follow-up period after surgery was 40 months (range 6-74 months).

The fistula was idiopathic in 218 patients (88%) and associated to inflammatory bowel disease (IBD) in 29 patients (11%), of which 26 associated to Crohn's disease (CD) and 3 to Ulcerative Colitis (UC).

Mean duration of symptoms before surgery was 18 months.

Patients who had an abscess preceding the fistula were 122 (52.8%) and, among them,77 (33.3%) underwent surgical drainage, and 45 (19.4%) drained spontaneously.

Twenty-seven patients underwent previous surgery for anal fistula in other hospitals prior to referral to our department. Fourteen patents had an history of previous anorectal surgery: haemorroidectomy (9 patients), lateral internal sphincterotomy (1), sphincteroplasty (1), Block repair of anterior rectocele (1), ileo-anal pouch (1), anal polyp excision (1).

Fifteen patients presented with proctological comorbidites: anal fissure (six patients), anal polyps (3), haemorrhoids (2), haemorrhoids plus polyps (2), fissure plus polyps (1) and anal condylomatosis (1). Patient with polyps and condilomatosis were treated at the same surgery.

On clinical examination, fifty-seven (23%) were intersphincteric, one hundred and fifty (60%) transphincteric, 2 (0.8%) suprasphincteric, 2 (0.8%) extrasphinteric, 7 (2.8%) recto-vaginal fistulas.

Five patients presented two or more fistula tracts and further three patients presented with multiple fistula tracts associated to abscess. In two cases a recto-vaginal fistula associated to another tracts was detected. One patients had necrotizing fascitis and one had a fistula with horseshoeing abscess.

The most common surgical procedure was seton placement (one hundred and sixty-two patients (65%) for the treatment of complex fistulae with involvement of sphincter apparatus and fistulae in Crohn's disease patients (table 1).

**Table 1:** Preoperative characteristics and treatment of patients presenting with simple anal fistula

		Second surgery	Overall
**NUMBER**	77 (31%)		

**FISTULA CLASSIFICATION**			

Inter-sphincteric	57 (23%)		

Mid/low trans-sphincteric	20 (8%)		

**PRINCIPAL SURGERY**			

Single staged fistulotomy	77 (31%)	8	85 (34%)

Postoperative Wexner score			

In order to drain the sepsis, loose setons were placed in 82 patients (table 2). While in 80 patients cutting setons were tight progressively in outpatients clinic.

**Table 2:** Preoperative characteristics and treatment of patients presenting with complex anal fistula

		2^nd ^surgery
**NUMBER**	162	

**FISTULA CLASSIFICATION**		

High Trans-sphincteric	122 (65%)	

Extra-sphincteric	2 (0.8%)	

Supra-sphincteric	2 (0.8%)	

Recto-vaginal	7 (2.8%)	

IBD	29 (11%)	

**PRINCIPAL SURGERY**		

Loose Seton	80 (32%)	11 ADF+permacol inj 17 ADF 18 LIFT 6 permacol injection

Cutting Seton	82 (33%)	8 (fistulotomy)

Postoperative Wexner score		

MEAN FOLLOW-UP (MONTHS)	40 (range 6-74)	

RECURRANCE RATE		

INCONTINENCE	2.4%	

Loose seton was placed initially in 11 patients and followed by mucosal advancement repair and Porcine dermal collagen matrix injection.

Eighty-five patients (34%) underwent fistulotomy; this procedure was the only surgery in 77 (31%) patients, mainly for intersphincteric and mid/low transphincteric fistulae. Moreover, fistulotomy was performed as part of treatment in eight patients with complex fistulae (8/80 = 10%).

Seventeen patients (6.8%) were submitted to mucosal flap advancement for recurrent idiopathic high transphincteric fistulae. Out of 17, seven had undergone one surgical procedure for fistula while ten had more than 2 procedures.

Eighteen patients underwent LIFT (ligation of intersphicteric tract). Six patients underwent permacol injection

At EUA seven (2.8%) patients had diagnosis of recto-vaginal fistula, and two of them presented more than 2 tracts. Five underwent loose seton placement followed by advancement flap repair and two abscess drainage plus seton.

The fistulae associated to ulcerative colitis were an horseshoeing fistula, treated with wide drainage and loose seton followed by mucosal advancement flap

In our audit, out of 26 Crohn's fistulae, 24 CD patients with anal fistula underwent loose setons. This was the only surgery in six cases. Seven patients had seton plus fistulectomy; five had abscess drainage and fistulotomy plus seton; five had abscess drainage plus seton; one patient had fistulotomy and fistulectomy plus seton. One had only fistulectomy, and the last CD patient had fistulotomy plus fistulectomy. Out of 26 CD patients, The remnant two CD patients had fistulotomy.

In one case, previously treated with mucosal flap for high transphincteric fistulae, a colostomy was performed because of a complete dehiscence of the flap.

Four cases were abscess drainage plus setons with or without fistulotomy for sepsis. Three patients underwent EUA-guided seton replacement. Two patients underwent fistula tract closure by fibrin glue injection. In a case fistulotomy was performed.

Three patients developed recurrence after seton placement for high transphincteric fistula; they were submitted to mucosal flap advancement with completely healing after seven weeks.

Two recurrences occurred after fistulotomy for intersphincteric fistula(2/85 = 2.3%); one patient was treated with fistulotomy, the other with cutting-seton placement.

Two patients submitted to mucosal flap advancement for recurrent high transphincteric fistulas were retreated with seton placement for anal sepsis. After three months, when sepsis was completely solved, they underwent a new mucosal advancement flap, with complete healing after 8-10 weeks.

In two CD patients, surgery was necessary three times. Two patients presenting with complex recto-vaginal fistula (> 2 tracts) underwent first fistulectomy and loose-setons, then two advancement flap plus Permacol injection. The other underwent firstly two abscess drainage and setons, then proctectomy for severe perianal disease with recto-urethral fistula.

Three cases of major faecal incontinence (1.3%) were detected. Two females with major faecal incontinence were observed during the follow up period; in a third CD patient major faecal incontinence was present in case of bowel movements higher than 4 daily.

In three cases (1.3%) minor incontinence was recorded and successfully treated with biofeedback in 2 cases and with permacol injection as bulking agent in one case.

The main treatment successfully eradicated the primary fistula track, in non IBD fistulae, in 151/247 patients (61%) and during the follow-up period all of whom remained healed at the time of last review. Two male patients still had a seton in situ controlling a residual primary fistula track.

## Discussion

Perianal fistulas have been a common troublesome pathology.

According to the Parks classification, the rate of intersphincteric fistulae reported in literature is 70%. Besides, 25% of fistulae are transphincteric, 5% are suprasphincteric and 1% extrasphincteric [14,15].

Differently, in our audit, 8% of patients presented with low transphincteric fistulae and about 23% of patients had a intersphincteric fistula. Moreover, more than 62% were complex fistulae and 2.8% were recto-vaginal, which required careful assessment of the treatment strategy.

According to the literature, in our clinical experience, surgical strategy was chosen according to type and complexity of the fistula, sphincter involvement presence of comorbidities and previous interventions.

Low transphincteric fistulas involve the lower third of external anal sphincter apparatusand are generally treated by fistulotomy with a high healing rate.

In our experience, eighty-five patients (34%) underwent fistulotomy; this procedure was the only surgery in 77 (31%) patients, mainly for intersphincteric and mid/low transphincteric fistulae without recurrences detected in the follow up period.

Besides, recurrence rate after fistulotomy in literature is noteworthy. In a recent retrospective trial on 624 patients, Garcia Aguillar reported a recurrence rate of 8% [5]. Factors associated with recurrence included type and extension of the fistula, lack of identification or lateral location of the internal fistulous opening, previous fistula surgery and the surgeon experience [5].

Differently, high fistulas with one or more tracts involving the upper external sphincter and levator ani remain a surgical challenge for the colorectal surgeon.

In this case, transrectal ultrasound to identify the tract and to define the anatomic relations with the muscles and the contiguous organs can be helpful, especially in conjunction with the use of peroxide, which delineates the tract on the ultrasound image. Magnetic resonance imaging (MRI) may also be helpful, especially in case of suprasphincteric and extrasphincteric fistulas [16,17].

According to our preoperative protocol, transrectal ultrasound and magnetic resonance imaging were not routinely performed. Besides, digital examination was an effective tool to assess fistula complexity ^3 ^and compared favourably with ultrasonography [18]. In this trial, ultrasonography and MRI were indicated mainly in the management of patients with Crohn's disease, in case of high fistulae with transphincteric, extrasphincteric, suprasphincteric and/or multiple tracks, recto-vaginal fistulae, often associated to proctitis.

Moreover, we didn't perform routinely preoperative anal manometry, advocated by some authors to prevent incontinence after fistula surgery, but without reliable results. Our thought is that manometry does not guarantee a protection against incontinente, but is more important the knowledge of risk factors, like previous fistula or perineal surgery, and findings during EUA.

In case of complex fistulas, the seton placement has been advocated either loose, to control infection, or cutting through the sphincter muscle gradually or as a bridge between two separate partial fistulotomies [19,20]. In our audit, the most common surgical procedure was the placement of seton (65%), usually applied in case of complex fistulae and Crohn's patients and if loose followed by flap procedure.

Regarding the comparison between seton and other techniques, Tang et al in a large multicenter Indian study (n = 503) comparing seton and fistulectomy, showed longer healing with chemical setons but lower recurrence rate (4% vs 11%) [21]^.^

Differently Zbar et al, compared conventional cutting setons vs 'internal anal sphincter preserving' cutting seton in 34 patients with trans-sphincteric fistulae and reported no statistically significant differences in recurrence (11.1% versus 6.25%), healing time (14 weeks versus 12 weeks) and continence impairment (5.5% versus 12.5%) [22].

Transanal advancement flap has been advocated as an effective treatment for trans-sphincteric fistulas passing through the upper or middle third of the external anal sphincter. In our cohort, 17 patients (6.8%) were submitted to mucosal flap advancement for recurrent idiopathic high transphincteric fistulae.

If we overview the literature, initially, the reported healing rates of flap repair varied between 84 and 100 percent [23,24]. Subsequently, during the last decade, several studies have revealed considerably higher recurrence rates.

Zimmerman et al, out of 87 patients, reported an healing rate of 67% after flap repair [25]. Similarly, Mitalas et al reported 68% healing rate after treatment of 80 patients with transanal advancement flap repair [26].

In our audit, the majority of the fistulae encountered were cryptoglandular (88%), nonetheless, the number of fistulae in patients with Crohn's disease was noteworthy (10.5%).

Fistulas in Crohn's disease are demanding to manage and resistant to many traditional approaches. The Crohn's fistulas are thought to originate as a deep penetrating ulcer in the anorectum, plugged with fecal material. Several conservative treatments have been described in the literature to manage Crohn's fistulas.

Medical therapy alone has been documented by several series with closure rate up to 50% [27].

Surgery for Crohn's fistulas has to be individualized to the patient's medical condition, the degree of activity of proctocolitis, together with the location and type of fistula.

Complex fistulas in CD patients should be treated conservatively to avoid the risk of incontinence. Seton placement is the gold standard in these cases and this helps the fistula to heal and allows continued drainage without abscess.

Only 6 recurrences were observed in patients without inflammatory bowel disease in our trial. Few reports of long-term follow-up and recurrence exist. In line with our results, Malouf [19] reported a 4% recurrence rate at 14 moths' follow up. A recurrence rate of 6.3% has been reported at a follow up of over 3 years by Vasilesky [28].

Faecal incontinence remains a problem after fistula surgery. Reported incontinence rates vary considerably from 0 to 40% largely because of lack of standardization and variable follow-up [19]. In the recent study of Garcia Aguillar [5], out of 624 patients, 45% complained of some degree of postoperative continence. Incontinence was associated with complex fistulas, type of surgery and previous fistula surgery. Besides in line with Malouf et al, in our audit, three cases of major faecal incontinence (1.2%) were detected and in three cases (1.2%) minor incontinence was recorded and successfully treated with biofeedback in 2 cases and with Permacol injection as bulking agent in one case.

## Conclusions

In summary, a high proportion of complex fistulas was seen in the present audit compared with previous studies. Despite this a satisfactory outcome was achieved in the vast majority with a relatively low rate of incontinence. Caution was used when dealing with anal fistula Crohn's disease, frequently complex and requiring several treatments and often treated with loose setons. New technologies provide promising alternatives to traditional methods of management particulary in case of complex fistulas. High-quality randomized control trials to evaluate the different surgical and non surgical treatment options are warranted.

## Competing interests

The authors declare that they have no competing interests.

## Authors' contributions

**PS **critical review. **FC,SD **and **EDL **manuscript preparation and critical review. **LF **and **GDVB **literature review and manuscript preparation. **VF **and **SMC **data collection and literature review. **GM **and **ALG **critical review. All authors read and approved the final manuscript.

## Pre-publication history

The pre-publication history for this paper can be accessed here:

http://www.biomedcentral.com/1471-230X/11/120/prepub
